# Pharmacoprophylaxis of alcohol dependence: Review and update Part I: Pharmacology

**DOI:** 10.4103/0019-5545.31514

**Published:** 2007

**Authors:** Sandeep Grover, Gaurav Bhateja, Debasish Basu

**Affiliations:** Drug De-addiction and Treatment Centre, Department of Psychiatry, Postgraduate Institute of Medical Education and Research, Chandigarh, India

**Keywords:** Alcohol dependence, disulfiram, acamprosate, naltrexone, topiramate

## Abstract

Alcohol dependence is a major problem in India. The pharmacological armamentarium for relapse prevention of alcohol has widened with the addition of new drugs. In this article, we review the pharmacology and efficacy of the four most important such drugs: disulfiram, naltrexone, acamprosate and topiramate. The first part of this two-part review series concerns the comparative pharmacology and the second part concerns the efficacy studies. Overall, all four of these drugs have modest but clinically significant usefulness as pharmacoprophylactic agents for relapse prevention or minimization of alcohol dependence. Combinations might be helpful, especially for naltrexone and acamprosate. The issue of supervision and compliance remains important, especially for such drugs as disulfiram and naltrexone. Topiramate is a promising new agent and requires further study. Disulfiram, while very effective in compliant patients, presents challenges in terms of patient selection and side effects. For patients with hepatic impairment, acamprosate is a good choice.

## INTRODUCTION

Alcohol dependence is a major problem in India. An estimated 34-42% of adult Indian population reports having used alcohol in their lifetime; 5-7% has been estimated to be abuser of alcohol and 10-20 million persons have been estimated to be in need of treatment for alcohol dependence, along with the steady rise in per capita alcohol consumption every year.[1] According to the Global Burden Report, alcohol accounted for 1.2% of total death and for 1.6 % of total disability adjusted life years in 1990 in India.[2] Besides the impact on the individual it also has an enormous impact on economy and public safety. Considering the impact of alcohol alone on the mortality and morbidity there is a definite need to treat this condition. On individual level the goal of treatment should be better functioning in all facets of life and on a societal level treatment should reduce crime, violence, family discord and other infectious and non-infectious diseases. Treatment involves detoxification followed by relapse prevention measures. To achieve the above stated goals most important facet of treatment is relapse prevention.

For many years, psychosocial methods such as group therapy and 12 step programs were the only effective therapies for the relapse prevention of alcohol dependence. Over the last 20 years, the role of adjuvant pharmacotherapy in optimising outcome in rehabilitation programmes for alcohol-dependent patients has become increasingly evident and over the years the pharmacological armamentarium for relapse prevention of alcohol has widened with the addition of new drugs. Further, now there is growing evidence that psychotherapy combined with pharmacological treatment is more efficacious than either alone.[3]

In recent years, many medications have been evaluated for the treatment of alcohol dependence, including those that interact with dopaminergic, serotonergic, opioid or glutamate and/or GABA systems. Disulfiram, naltrexone and acamprosate are currently the only treatments approved for the management of alcohol dependence. Other drugs which have been used for the same are calcium carbide, selective serotonin reuptake inhibitors, tiapiride, lithium, nalmefene, metronidazole, ondansetron and topiramate.

Of the three approved drugs for relapse prevention of alcohol dependence, the oldest and best known is disulfiram. Naltrexone was launched in India in 1998, which is a muopioid receptor antagonist traditionally used for opioid dependence, but has also been shown to be beneficial for alcohol dependence. The new addition is acamprosate, launched in 2002 in India, which is a drug with the specific and exclusive indication for alcohol pharmacoprophylaxis. Most recently topiramate has been promoted for pharmacoprophylaxis of alcohol dependence.

The development of treatments for alcohol dependence has been significantly complicated by the multiple actions of ethanol at the neurotransmitter level, heterogeneity among patients with alcohol dependence, the complexity of defining and measuring the phenomenon of craving and the challenge of quantifying alcohol intake in patients. With the availability of these drugs it is now important to review the literature concerning the use, indication and efficacy of these drugs. In this article we will review the mechanism of action, compare the pharmacokinetics and pharmacodynamics, indications and contraindications, precautions (Part I of this review) and the evidence of efficacy for disulfiram, naltrexone, acamprosate and topiramate (Part II of this review).

## DISULFIRAM

The pharmacokinetic and pharmacodynamics profile of disulfiram is given in Table 1. Disulfiram irreversibly inhibits aldehyde dehydrogenase (the enzyme that converts the relatively toxic metabolite acetaldehyde to the benign metabolite acetate); which is necessary for the metabolism of ethanol.[4] Ingestion of a single dose begins to affect ethanol metabolism within 1 to 2 hours; its peak effects are seen at 12 hours and its sustained effects (usually 12-72 hours) depend on the rate of new enzyme synthesis. In some individuals, the effects of a single dose can last up to 2 weeks.[5] In the body disulfiram is converted into diethylthiomethylcarbamate (Me-DTC) through 3 intermediate steps. Me-DTC is considered to be most powerful irreversible inhibitor of aldehyde dehydrogenase.[6] If ethanol is ingested while taking disulfiram, the inhibition of aldehyde dehydrogenase causes increased levels of acetaldehyde; this results in a toxic reaction, commonly known as disulfiram-ethanol reaction (DER). Symptoms include throbbing headache, flushing, dizziness, nausea, vomiting, blurred vision, hypotension, tachycardia and hyperventilation. In addition, significant cardiovascular effects (e.g., chest pain, palpitations, tachycardia and hypotension) can occur. Severe reactions (which arise when disulfiram is used at very high doses or in individuals with cardiovascular disease) can include myocardial infarction, arrhythmia, congestive heart failure (CHF), or death. Usually the symptoms of DER last for 30-120 min, depending upon both on dose and amount of ethanol consumed.[7,8] The threshold for the reaction is approximately 7 ml of 100% ethanol or its equivalent.[9] Serious clinical sequalae of disulfiram-ethanol reactions (e.g., shock, hypotension, or myocardial ischemia) should be managed aggressively; there is no specific antidote to the disulfiram-ethanol reaction. The supportive management includes putting the patient in trendelenburgs position and infusing isotonic saline with constant monitoring of blood pressure. Antihistaminics have been shown to partially block some of the symptoms.[7]

**Table 1:** Comparison of pharmacology of disulfiram, naltrexone and acamprosate

	Disulfiram	Naltrexone	Acamprosate	Topiramate
T^½^	7.3 hrs	4 hrs (1.1-10 hrs)	13hrs	21 hrs (19-23 hrs)
Metabolite	Diethylthio methyl carbamate (Me-DTC), t^½−^ 11.2hrs	(β-naltrexol, t^½^ = 12 hrs.	No metabolite	6 metabolites
Peak plasma level	-	60-90 min.	5.2 hrs	2 hrs
Oral bio availability	80-90%	5-40%	11%	80%
First pass metabolism	+++	+++	No	+
Plasma protein binding	96%	20%	No	9-17%
Metabolism	Reduced by glutathione reductase of erythrocytes, In liver undergoes glucuronidation conjugation and oxidation	Reduction, glucuronidation and conjugation through liver	Not metabolized	Six metabolites formed via hydroxylation, hydrolysis, and glucuronidation have been identified in humans, none of which constitutes more than 5% of an administered dose.
Elimination	Kidney = 65%; Faeces = 20%; Lungs	Kidney	Kidney	> 80% through kidney
Dose	200-300 mg/day (250 mg)	50 mg/ day	> 60 kg = 333x6 tab; <60kg = 333 × 4tab	200-400 mg/day
Side Effects	Skin -Rash, Pruritus, Dermatitis Psychiatric –Anxiety, Depression, Psychosis **CNS** – Neuropathy, convulsions, Confusion, Paraesthesia Gastrointestinal – Hepatitis, Metallic taste, Abdominal upset	Gastrointestinal – Nausea, Vomiting, Constipation, Heart burn, Hepatic enzyme elevation	Gastrointestinal – Diarrhea, Mild abdominal pain	**CNS**- somnolence, dizziness, ataxia, speech disorders and related speech problems, psychomotor slowing, abnormal vision, difficulty with memory, parasthesias and diplopia
		**CNS** –Nervousness, Agitation, Headache, Insomnia Musculoskeletal -Joint pain, Muscle soreness	Skin - Pruritus CNS - Confusion Others - Decrease libido	
Investigation for start of therapy	Liver function test, ECG	Liver function test, Urine TLC	Renal function test, Liver function test	Renal function test
Monitoring	Liver function tests – Baseline, then at 2 wks interval for first 2 months, then 3-6 months interval Blood count-3-6 months	Liver function tests		Renal function test
Absolute contraindications	H/o allergic reaction to drug, Pregnancy, poor motivation, suicidal, impulsive	H/o allergic reaction, currently dependent on opioids, Severe liver disease	H/o allergic reaction	H/o allergic reaction
Relative contraindications	Poor cardiovascular reserve, Abnormal liver tests, Psychosis, Derange renal functions, Peripheral neuropathy, Seizure disorder	Pregnancy, Lactation, Chronic pain, Children	Severe liver disease, Renal insufficiency (Cr>l. 2), Pregnancy, Lactation, <18 yr>65 yrs	
Drug interactions	Inhibitor of P450: increase the Plasma level of MAOI, Barbiturates, Benzodiazepine, Amitriptyline, Imipramine, Warfarin, Phenytoin of opioid analgesics	Increase plasma levels of Thioridazine, Blocks the action of opioid analgesics	Inactivation of tetracycline by calcium component	Enzyme inducers (Carbamazepine, Phenytoin) decrease the concentration of topiramate. Topiramate reduces levels of digoxin, oral contraceptives.
Abstinence for starting treatment	12 hours after last drink	2-7 days after last intake	2-7 days after last intake	Can be used for withdrawal symptoms also
Effects craving	-	±	++	+
Recommended treatment duration	One year	6-9 months	One year	
Motivation for abstinence	++++	+	++	
Cost of therapy/day	1.60 Rs/-	40-50 Rs/-	20-30 Rs/-	
Advantage	Low cost, OD dose	Anticraving, OD dose, Useful for opioid and Nicotine	Min. interactions with drugs, useful in hepatic impairment	
Disadvantages	High motivation for abstinence, Aversive therapy, S/E and interactions	Costly, Side effects, Interactions	Cost, TID dosage	

Besides the pharmacological action disulfiram also acts psychologically. The various psychological actions include deterrence, autosuggestion, therapeutic rituals around, renewed active thinking process and continuous reinforcement of a sober lifestyle and development of new coping skills. Simply speaking disulfiram enables patients to expose themselves to alcohol-related cues and environments while the usual alcohol-drinking response is prevented and it facilitates the practice and development of alternative, alcohol-free coping techniques.[10] High doses of disulfiram have also been reported to inhibit cerebrospinal dopamine β-hydroxylase in rats.[11,12] The result with therapeutic dose to man vary.[13,14] Patients with very low activity of dopamine hydroxylase appear to be sensitive to disulfiram in the sense that they may develop a transient psychotic condition, probably because of an increased ratio between dopamine and noradrenaline in the brain.[13]

The usual dose of disulfiram is 250 mg/day. Doses less than this tend not to produce aversive reactions with alcohol ingestion, while doses greater than (or equal to) 500 mg/day are associated with higher rates of problematic side effects (e.g., psychosis, hypertension and hepatitis).[5] Common side effects include gastrointestinal side effects (such as nausea and dyspepsia), mild sedation and a metallic or garlic taste. Disulfiram can cause hepatitis (usually dose-related and quite rare at 250 mg/day); therefore, liver function tests (LFTs) should be checked prior to starting treatment (most practitioners consider it acceptable to initiate treatment with disulfiram if the LFTs are less than twice the upper limit of the normal value). LFTs should be checked every 2 weeks for the first 2 months, then roughly every 3-6 months while disulfiram therapy continues.[15] In the literature, there are no clear guidelines about treating patients with early cirrhosis. In general, if patients have early cirrhotic changes but their liver enzyme levels are less than three times the normal, disulfiram can be started at lower doses. Among the side effects, it has been found that the most common side effects are that of hepatic (34%), followed by neurological (21%), cutaneous (15%) and others (26%).[16] Drug interactions of disulfiram must be remembered while prescribing. Disulfiram inhibits the biotransformation of warfarin, phenytoin, isoniazid, some benzodiazepines (e.g., diazepam) and tricyclic antidepressants (TCAs) (e.g., desipramine and imipramine). Therefore, prothrombin time (PT) should be monitored in patients taking warfarin and levels of phenytoin and TCAs should be monitored if patients are taking these medications in combination with disulfiram.[16]

As with all medications, the risks of treatment must be balanced against the risks of no treatment (in this case, the health risks of continued, untreated alcohol use). Disulfiram is relatively contraindicated when patients have coronary artery disease (CAD), chronic cardiac arrhythmias, cardimyopathy with CHF, chronic renal failure, cerebrovascular disease and severe pulmonary disease. Furthermore, disulfiram use is contraindicated in pregnancy (because its use is associated with birth defects). Disulfiram can also exacerbate peripheral neuropathy and psychosis and it can lower seizure threshold. Furthermore, individuals who are unable to understand the consequences of using a substance that adversely reacts with disulfiram (e.g., patients with limited intelligence or with an organic brain syndrome) should not be prescribed disulfiram. Finally, patients should not receive disulfiram if they have a history of an adverse neurologic, psychiatric, or cardiovascular reaction to disulfiram. Disulfiram should also be avoided in patients with a history of dangerous impulsive behavior or significant suicidality.[17]

## NALTREXONE

It is an opioid antagonist and has been used for relapse prevention for alcohol, after the elucidation of role of endogenous opioids in alcohol dependence. Animal and human experimental studies have shown that acute administration of alcohol stimulates the release of endogenous opioids such as β-endorphins.[18] Opioid antagonists have also been shown to decrease alcohol consumption in several animal species and humans.[19] Based on this, several theories have been proposed how endogenous opioids and opioid antagonists are related to alcohol consumption. These include opioid surfeit hypothesis, opioid compensation hypothesis and opioid response hypothesis. According to opioid surfeit hypothesis alcohol consumption increases endogenous opioid activity, which in turn leads to additional and eventually, excessive drinking. Opioid compensation hypothesis states that propensity to drink alcohol is biologically determined and also related to under activity of endogenous opioid systems. Alcohol like, stress increase activity in central and peripheral system, which compensate for endogenous opioids deficiency, but also stimulates drinking. When alcohol consumption stops, there is a rebound deficiency in opioid activity, which eventually causes drinking to resume. The third hypothesis, opioid response hypothesis presumes that the basal levels of opioid activity do not have effect on alcohol consumption. Shortly after alcohol consumption there is increase in endogenous opioid activity that mediates positive reinforcing effects and stimulates consumption of more alcohol.[20]

The pharmacokinetic and pharmacodynamics profile of naltrexone is given in Table 1. The usual dose of naltrexone used in the treatment of alcohol dependence is 50 mg/day. It is rapidly and completely absorbed following oral administration and the oral bioavailability is between 5-60%.[21] Naltrexone undergoes extensive first-pass metabolism in the liver to β-naltrexol and only 5% of the drug reaches the systemic circulation.[21] Although a much weaker antagonist than naltrexone, the half-life of β-naltrexol is longer and plasma concentrations of the metabolite are always higher than those of the parent drug. The mean elimination half-life values for naltrexone and 6-β-naltrexol are four hours and 13 hours, respectively.[22] Before initiating treatment, it is essential to assess the patient for recent opioid use (within the past 7-10 days) to avoid precipitating opioid withdrawal.[23] Before starting naltrexone, baseline LFT should be done and follow-up LFT should be done after 1 month and if the results are acceptable, then LFTs should be done at every 3 and 6 months after the initiation of treatment, depending on the severity of liver dysfunction at the start of treatment. More frequent monitoring is indicated for cases in which dose adjustments are being made, baseline LFTs are high, there is a history of hepatic disease, disulfiram or other potential hepatictoxic medication is added to the treatment. Naltrexone should not be used in patients who have acute hepatitis or liver failure. Some authors recommend that naltrexone be avoided in patients with aspartate transaminase levels three times greater than normal, while others set the limit at five-time normal.[22,24] Common adverse effects, which may include nausea, headache, depression, dizziness, fatigue, nervousness, insomnia, vomiting and anxiety, occur at the initiation of treatment in approximately 10 percent of patients.[25–27] Unlike disulfiram, naltrexone does not appear to alter the absorption or metabolism of alcohol and does not have major adverse effects when combined with alcohol. Some patients, however, have noted increased nausea caused by drinking alcohol while taking naltrexone. Patients on naltrexone are less likely to relapse to heavy drinking following a lapse in abstinence.[28] Naltrexone is usually contraindicated in patients with hypersensitivity to naltrexone, patients actively dependent on opioids, patients receiving opioid containing medications, patients who have acute hepatitis or liver failure. Data regarding use of naltrexone in pregnancy, lactation and children is not available; hence it should be used only when the potential benefits exceed the risks.[29] The physician should be cautious when combining naltrexone with other drugs associated with potential liver toxicity, such as acetaminophen and disulfiram. Naltrexone also interacts with thioridazine and oral hypoglycemics.[30]

## ACAMPROSATE

The pharmacokinetic and pharmacodynamics profile of acamprosate is given in Table 1. The precise mechanism of action of acamprosate remains to be elucidated. Chronic exposure to alcohol causes increase in activity of the excitatory glutamate system and a decrease in the inhibitory gamma-aminobutyric acid (GABA)-ergic system in the central nervous system.[31] Even after stopping the alcohol intake due to increase in the glutamate activity, the central nervous system remains hyperexcitable and leads to withdrawal symptoms. The neuronal readaptation process requires years or more of abstinence to resolve.[32] Acamprosate, which has a similar structure to GABA, acts at both NMDA (glutaminergic) and GABA receptors to normalize the glutaminergic excitation associated with alcohol withdrawal and with early abstinence. Recent data suggest that acamprosate may interact with a polyamine site on the NMDA receptor complex and differentially modulate the activity of the receptor on the basis of existing polyamine levels.[33–35] In addition, there is evidence of action at an additional binding site on the metabotropic mGluR5 receptor;[36] this receptor has been implicated in addictive processes, modulates glutaminergic neurotransmission and interacts with the NMDA receptor. It is likely that these effects of acamprosate on the polyamine binding sites and mGluR5 receptors attenuate glutaminergic hyperexcitability.[37,38] Analyses of alcohol intake patterns in studies demonstrating acamprosate-induced reductions in drinking behavior have shown that acamprosate does not reduce the motivation to obtain ethanol or delay the initiation of drinking, but rather decreases ethanol preference and continued intake after a normal onset of drinking, i.e., after alcohol and acamprosate have been experienced in combination.[39–41] Interestingly, it has been reported recently that part of acamprosate's clinical efficacy is due to a reduction in the severity of, rather than the latency to, relapse in alcoholics trying to abstain.[42]

Absorption of acamprosate through gastrointestinal system is slow and sustained with significant variation from person to person. Most of the drug is absorbed in first 4 hours and the maximum plasma concentration is achieved after 5.2 hours. The bioavailability of acamprosate is only 11%. Concomitant food intake (but not alcohol) decreases the bioavailability by 20%. Steady state plasma concentration is achieved after 7 days of starting acamprosate. The t ½ is about 13 hours. It is mostly (90%) excreted unchanged in urine and it is recommended that in patients with severe renal impairments, dose should be reduced. The pharmacokinetics of acamprosate is not affected by hepatic impairment and is therefore suitable for many patients with liver dysfunction. The common side effects reported with acamprosate are diarrhoea, abdominal pain, nausea, vomiting, Pruritus, dizziness, confusion, drowsiness and headache. Some patients may report maculopapular rash, bullous skin lesions and fluctuation in libido. Usually the side effects are mild and transient and resolve on dosage reduction or withdrawal. Much of the drug interactions of acamprosate have not been evaluated. No adverse interaction has been reported in human volunteers, when used in combination with diazepam, oxazepam, phenobarbital, meprobamate, imipramine and disulfiram.[43]

Acamprosate is available in 333 mg tablets. The recommended dosage in patients with body weight less than 60 kg is 4 tablets per day and for those with body weight more than 60 kg is 6 tablets per day. The tablets should be given in three divided doses. The recommended duration for treatment is 1 year. Contraindications to acamprosate includes hypersensitivity to the drug, pregnancy, breast feeding, renal impairment (serum creatinine > 0.12 mmol/liter) and severe hepatic impairment. It should not be administered to children or the elderly.[43]

## TOPIRAMATE

Topiramate is a sulfamate-substituted analog of fructose-1, 6, -diphosphate that was originally synthesized as a potential antidiabetic agent acting by inhibiting gluconeogenesis.[44] Its structural similarity to acetazolamide, which has anticonvulsant properties prompted its testing as an anticonvulsant; which was later found to be true.[45,46] Johnson in 1996 for the first time hypothesized that topiramate would be a promising medication for the treatment of alcohol dependence because of its dual action to facilitate GABA-A mediated inhibitory impulses and at the same time antagonizing AMPA and kainate glutamate receptors, resulting in suppression of ethanol-induced nucleus accumbens dopamine release, thereby inhibiting the reinforcing effects of alcohol associated with its abuse liability. Later on various other mechanisms of action were proposed to be helpful in treating alcohol withdrawal symptoms also.[47]

The pharmacokinetic and pharmacodynamics profile of topiramate is given in Table 1. Most of the pharmacokinetic and pharmacodynamics properties have been studied in epileptic subjects. It is absorbed rapidly and nearly completely, with peak plasma concentration occurring after about 2 hours after a dose of 400 mg.[48] The absorption is linear across wide ranges of doses.[49] Food affects the rate of absorption of topiramate, but not the extent.[50] Only 9-17% of the drug is bound to plasma proteins.[49] In contrast to the other antiepileptic drugs topiramate is not metabolized extensively by the liver when administered alone. In the absence of hepatic enzyme induction, >80% of a single dose is excreted unchanged in the urine.[51] When given along with hepatic enzyme inducers about 40-50% of the dose is metabolized.[52,53] In the absence of enzyme induction mean plasma elimination half life is 19-23 hour and in the presence of the enzyme inducers plasma half life is reduced to 12-15 hours.[54] In vitro studies have shown that topiramate inhibits CYP2C19.[55] The plasma concentration of topiramate is reduced by 40% and 48%, when coadministered with carbamazepine and phenytoin respectively.[52,53] When administered with valproate the plasma concentration of topiramate reduces by 17% and concentration of valproate increases by 11%.[56] When given with drugs like oral contraceptives and digoxin, topiramate reduces their levels.[57,58] The usual dose of topiramate is 200-400 mg/day. The initial dose is 25-50 mg a day, increased by 25-50 mg a day each week as needed until the maximum of 400 mg a day. Usually it is given in two divided doses. The commonest adverse events across the studies are paraesthesias/numbness, nausea and vomiting, cognitive impairment, headache, dizziness, sedation/drowsiness, fatigue, decreased appetite, frequent peristalsis, somnolence, blurred vision, slow memory.[59] There is one reported case of psychotic features,[60] a case of delirium in a patient who overmedicated with 800 mg of topiramate and tranylcypromine sulphate (170 mg) combined with alcohol,[61] a case of acute narrow angle glaucoma[62] and 2 cases of haematuria.[63] Rare but serious adverse events documented with the use of topiramate include metabolic acidosis, acute myopia, acute glaucoma, oligohidrosis and hyperthermia.[59]
